# Cysteine Depletion Causes Oxidative Stress and Triggers Outer Membrane Vesicle Release by *Neisseria meningitidis*; Implications for Vaccine Development

**DOI:** 10.1371/journal.pone.0054314

**Published:** 2013-01-23

**Authors:** Bas van de Waterbeemd, Gijsbert Zomer, Jan van den IJssel, Lonneke van Keulen, Michel H. Eppink, Peter van der Ley, Leo A. van der Pol

**Affiliations:** 1 Vaccinology, National Institute for Public Health and the Environment (RIVM), Bilthoven, The Netherlands; 2 Bioprocess Engineering, Wageningen University, Wageningen, The Netherlands; Tulane University School of Medicine, United States of America

## Abstract

Outer membrane vesicles (OMV) contain immunogenic proteins and contribute to *in vivo* survival and virulence of bacterial pathogens. The first OMV vaccines successfully stopped *Neisseria meningitidis* serogroup B outbreaks but required detergent-extraction for endotoxin removal. Current vaccines use attenuated endotoxin, to preserve immunological properties and allow a detergent-free process. The preferred process is based on spontaneously released OMV (sOMV), which are most similar to *in vivo* vesicles and easier to purify. The release mechanism however is poorly understood resulting in low yield. This study with *N. meningitidis* demonstrates that an external stimulus, cysteine depletion, can trigger growth arrest and sOMV release in sufficient quantities for vaccine production (±1500 human doses per liter cultivation). Transcriptome analysis suggests that cysteine depletion impairs iron-sulfur protein assembly and causes oxidative stress. Involvement of oxidative stress is confirmed by showing that addition of reactive oxygen species during cysteine-rich growth also triggers vesiculation. The sOMV in this study are similar to vesicles from natural infection, therefore cysteine-dependent vesiculation is likely to be relevant for the *in vivo* pathogenesis of *N. meningitidis*.

## Introduction

The release of outer membrane vesicles (OMV) is observed among many bacterial species including gram-negative pathogens like *Escherichia coli*, *Vibrio cholerae*, *Salmonella typhimurium* or *Neisseria meningitidis* [1]. Pathogens produce OMV for *in vivo* survival, virulence or interactions with the host immune system [2], [3], [4]. The vesicles are spherical particles with a diameter of 50–200 nm, containing a phospholipid bilayer with outer membrane proteins, lipopolysaccharide (LPS or endotoxin) and a lumen with periplasmic constituents [2], [5]. *N. meningitidis* serogroup B epidemics in Norway, Cuba and New-Zealand were successfully controlled with outer membrane vesicle vaccines, which was a milestone for the application of OMV in vaccinology [6], [7], [8], [9]. These vaccines however required extraction with a detergent (deoxycholate), to decrease the amount of toxic LPS. The detergent-extraction was effective but resulted in partially intact and aggregated vesicles with an altered composition [10], [11], [12]. Discovery of the *lpxL1* mutation successfully attenuated the LPS of *N. meningitidis* and allowed the use of detergent-free OMV [13]. This provided vaccine concepts with equally low toxicity but improved immunological and biochemical properties [11], [12], [14], [15]. Detergent-free OMV are more similar to *in vivo* produced vesicles [5] and are now used for the development of several next-generation vaccines against serogroup B meningococcal disease [16], [17], [18], [19], [20].

There are two types of detergent-free OMV. The first type, sOMV (spontaneously released OMV), is as similar to *in vivo* vesicles as possible with a production system for human vaccines [5]. The sOMV are released by the bacterium during *in vitro* growth, without any treatments to enhance vesiculation [21], [22]. The second type, eOMV (extracted OMV, also referred to as native OMV) [15], [17], [23]), uses detergent-free extraction with ethylene-diamine-tetraacetic acid (EDTA) to chelate calcium and magnesium ions. This destabilizes the outer membrane which enhances eOMV release and preserves the desired biochemical and immunological properties, even though eOMV are not fully identical to sOMV [11], [24], [25], [26], [27]. The properties do change significantly when detergents are added to the extraction buffer. The purification of eOMV is less straightforward than sOMV purification due to more complicated handling (i.e. the extraction step; Figure S1). New approaches that release sOMV in sufficient quantities to compensate the EDTA extraction step are therefore relevant for vaccine development.

Even though the existence of sOMV is known for decades, the mechanism that triggers their release is not fully understood [28], [29]. A model was proposed for *S. typhimurium* in which the density of associations between inner membrane, outer membrane and peptidoglycan layer regulates vesicle release [2]. This model was in agreement with observations that disruption of transmembrane anchor genes increased vesiculation (i.e. *tol*, *pal* and *ompA* in *E. coli* or *rmpM* in *N. meningitidis*) [11], [30]. Not only physical changes to the outer membrane but also external stimuli can trigger sOMV release. For *E. coli* these stimuli include heat shock, activitation of the envelope stress response pathway or lysine depletion [31], [32], [33], [34]. Such external triggers have not been identified for *N. meningitidis* but recent work indicated that vesicle yield increased significantly during stationary growth [23].

This study demonstrates that a novel external stimulus, depletion of cysteine, can trigger the onset of stationary growth and sOMV release by *N. meningitidis*. It is substantiated that cysteine depletion causes oxidative stress as the intracellular signal for increased vesiculation. The results also demonstrate that this approach is applicable for vaccine production.

## Results

### Cysteine is the Growth-limiting Medium Component

**Figure 1:**
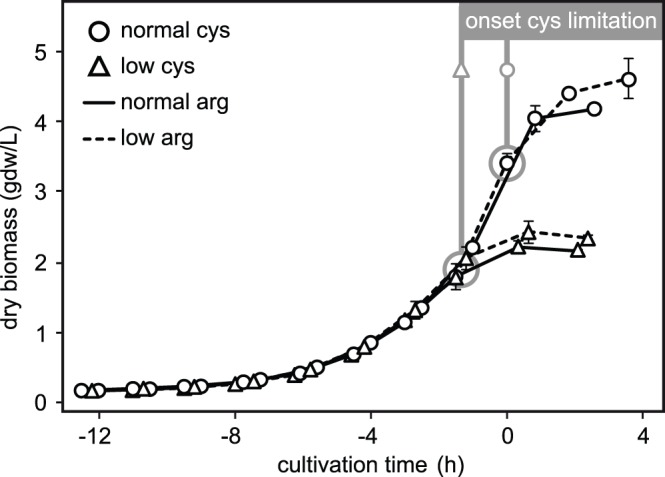
Cysteine is the growth-limiting medium component. Previous work demonstrated that vesicle release by *N. meningitidis* increased during the stationary growth phase [23]. Nutrient analysis of the chemically defined medium indicated that only arginine and cysteine were depleted during early stationary growth. Therefore, concentrations of both components were systematically lowered to identify the growth-limiting nutrient. Black lines and black intersected lines represent growth curves on media with normal and low arginine concentration, respectively. Normal and low cysteine concentrations are marked with circles and triangles. Cultivation time t = 0 represents the expected onset of stationary growth on reference medium with normal amounts of cysteine and arginine. The results indicate that cysteine, not arginine is the growth-limiting component. See Figure S2 for corresponding nutrient data.

Previous work showed that vesicle release by *N. meningitidis* increased during the stationary growth phase [23]. To identify the growth-limiting medium component of these experimental conditions, nutrient consumption was monitored. Arginine and cysteine were depleted upon onset of stationary growth, while the growth medium still contained sufficient amounts of all other components (data not shown). Therefore, concentrations of arginine and cysteine were lowered systematically to identify the growth-limiting component (Figure 1). Control medium with normal amounts of cysteine and arginine produced regular growth curves in shake flasks (exponential growth; dry biomass yield 3.9±0.1 gdw/L). Medium with normal cysteine but half the normal amount of arginine gave identical growth (exponential; 4.2±0.3 gdw/L), indicating that less arginine did not have an effect on growth. Media with half the amount of cysteine however produced half the amount of biomass, regardless the amount of arginine (1.9±0.1 gdw/L and 2.1±0.1 gdw/L for normal and low arginine, respectively). Nutrient analysis confirmed that cysteine was indeed depleted upon onset of stationary growth for all 4 media, while arginine depletion ocurred at a different time point (Figure S2). These results indicate that cysteine, not arginine, is the growth-limiting component of the medium.

### Transcriptome Analysis Identified 149 Cysteine Regulated Genes

Transcriptome analysis was performed with DNA microarrays to identify genes that are regulated after cysteine depletion. Growth and cysteine availability of triplicate cultivations on normal growth medium were monitored. Transcriptome analysis was performed on samples at −2.5, −1.4 and −0.4 hours (before cysteine depletion) and at +1.4 and +3.5 hours (after cysteine depletion). Principal component analysis on the full transcriptome confirmed high reproducibility of replicates (Figure S3A). The experimental treatment (cysteine depletion) accounted for 70% of total gene expression variation and resulted in 2 distinct groups on the first principal component axis (PC1; corresponding to ‘before’ and ‘after’ cysteine depletion). PC2 explained 7% of all variation and the other principal components <5%, indicating that additional effects had only a minor impact on overall gene expression. Subsequent statistical analysis selected 149 significantly regulated genes (Fold Ratio >2.0 and False Discovery Rate <0.10). Upon cysteine depletion, 90 genes were upregulated and 48 were downregulated. A variable expression pattern was observed for 11 genes (Figure S3B).

### The Gene Expression Profile of Cysteine Depletion Resembles (Oxidative) Stress and Virulence

**Figure 2:**
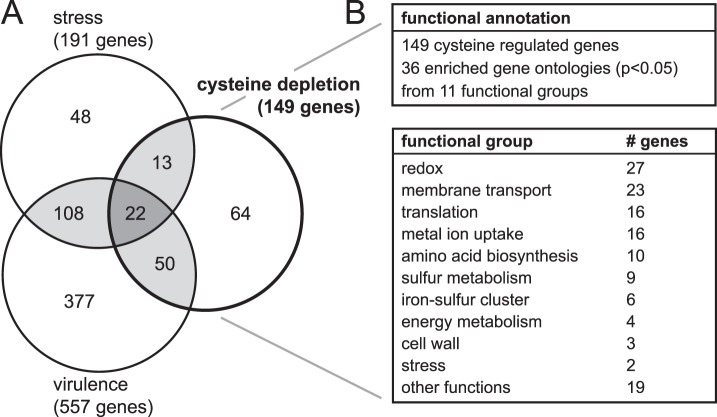
The gene expression profile of cysteine depletion resembles (oxidative) stress and virulence. Intracellular effects of cysteine depletion were explored with transcriptome analysis, resulting in 149 significantly regulated genes. (A) Significant overlap is found (p<0.0001) between cysteine regulated genes and relevant literature (compiled gene sets ‘stress’ [35], [36] and ‘virulence’ [37], ), indicating that similar mechanisms are involved in these different stimuli. (B) Functional annotation of cysteine regulated genes identified 36 enriched Gene Ontologies (p<0.05) from 11 functional groups. In addition to expected groups like sulfur metabolism or translation, several functional groups are related to oxidative stress (i.e. redox or iron-sulfur cluster). Therefore it is hypothesized that cysteine depletion impaires the sulfur supply for iron-sulfur protein biogenesis, ultimately resulting in oxidative stress. Details of the transcriptome analysis are provided in Figure S3 (principal component analysis and gene clustering) and Table S1 (expression data with functional annotation).

Cysteine regulated genes were compared with available literature. Genes from a heat shock study [35] had significant overlap with peroxide stress [36] (p<0.0001; binomial distribution probability). Since both studies investigated stress stimuli they were compiled into a single group (‘stress’) for the comparison with cysteine depletion. In addition, studies on iron depletion [37] and two host interaction studies [38], [39] were compiled to ‘virulence’ based on significant overlap (p<0.0001). Studies on restrictive oxygen conditions [40] and putative pathogenicity genes [41] were not used due to insufficient overlap with cysteine regulated genes and/or available literature. The compiled gene sets ‘stress’ and ‘virulence’ were then compared with the cysteine depletion genes, revealing a significant overlap between the 3 groups (p<0.0001). The overlap indicates that similar mechanisms may be involved in these biological events (Figure 2A). Functional annotation of 149 cysteine regulated genes revealed 36 enriched Gene Ontologies (p<0.05), related to 11 functional groups (Figure 2B). Functional groups with distinct expression patterns were sulfur metabolism (9/9 genes upregulated), iron-sulfur cluster (6/6 upregulated), translation (13/16 downregulated), metal ion uptake (11/16 upregulated), cell wall (3/3 downregulated) and stress (2/2 upregulated). Non-distinct patterns were observed for redox (27 genes), membrane transport (23 genes), amino acid biosynthesis (10 genes), energy metabolism (4 genes) and other functions (19 genes). Some functional groups were directly related to the experimental context, like sulfur metabolism and amino acid biosynthesis (cysteine depletion), downregulated translation machinery (stationary growth) or membrane transport (upregulated sulfate uptake genes). The remaining functional groups were primarily related to oxidative stress (redox, iron-sulfur cluster, metal ion uptake and stress). Details are listed in Table S1.

### Cysteine Depletion Triggers Increased sOMV Release

**Figure 3:**
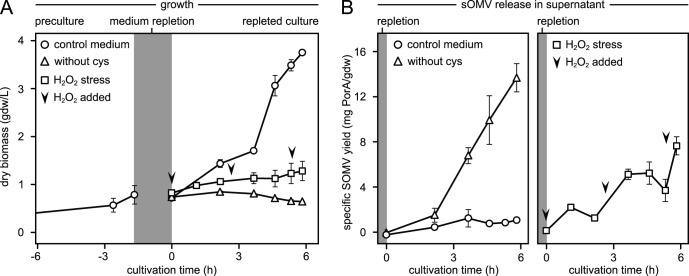
Cysteine depletion triggers sOMV release. The effect of cysteine depletion on sOMV release is demonstrated with a repletion experiment in shakeflasks. (A) Pre-cultures are grown on control medium with cysteine (circles). Harvested cells are repleted with control medium (circles) or medium without cysteine (triangles). Shake flasks without cysteine enter the stationary phase, while cysteine repleted flasks continue growth normally. A third group (squares) is repleted with control medium, but growth is repressed with peroxide to mimic oxidative stress and verify the transcriptome data (arrows indicate peroxide additions). Y-axis represents dry biomass concentration (g dry weight per L). (B) Vesicle release in the cultivation supernatant is monitored with a fluorescent probe up to 6 hours after repletion. Shake flasks with control medium grow normally but do not release sOMV, while cysteine depletion triggers a sustained release of vesicles. Oxidative stress has a similar but transient effect, since sOMV are released temporarily after each peroxide addition. Cysteine depletion is therefore an external stimulus for sOMV release, which induces oxidative stress as the intracellular signal. Y-axis represents specific sOMV yield (mg PorA antigen per g dry weight).

Based on the transcriptome results it was hypothesized that in addition to growth arrest, cysteine depletion causes oxidative stress as an intracellular signal for sOMV release. The hypothesis was verified with a medium repletion experiment in shake flasks (Figure 3). Precultures on control medium with the normal amount of cysteine were pooled and repleted with control medium or medium without cysteine (two groups). A third group was repleted with control medium but growth was repressed with peroxide to induce oxidative stress. Repleted cultures were monitored for growth and sOMV release. The sOMV were measured in small culture supernatant samples (sterile filtered; <3 mL) with a fluorescent probe that specifically binds phospholipid bilayer structures. Biomass concentration of the pooled preculture (0.78 gdw/L) was comparable to the biomass density directly after repletion (0.76±0.05 gdw/L), indicating that no biomass losses ocurred during the repletion procedure. Shake flasks that were repleted with control medium continued growth to a final dry biomass concentration of 3.75±0.02 gdw/L. Cysteine depleted and oxidative stress shake flasks did not grow, resulting in final biomass concentrations of 0.64±0.01 and 1.28±0.20 gdw/L, respectively (Figure 3A). The sOMV results however revealed opposite effects. Control shake flasks grew normally but had a low specific sOMV yield of 1.1±0.2 mg PorA antigen/gdw, while the cysteine depleted shake flasks yielded 13.7±1.3 mg PorA/gdw (with a constant sOMV release rate). Oxidative stress shake flasks temporarily released sOMV after each peroxide addition (variable sOMV release rate) resulting in an intermediate yield (7.7±0.8 mg PorA/gdw; Figure 3B). Shake flasks were harvested before cysteine in the control medium was exhausted, to prevent unintended sOMV release. Integrity of sOMV in the culture supernatant was confirmed with dynamic light scattering (DLS), which measured a distinct peak around 100 nm indicating that the bacteria were not desintegrating while releasing the OMV (data not shown). The above results demonstrate that cysteine depletion triggers increased sOMV release. Other stress stimuli like growth repression with peroxide can mimic this effect.

### Cysteine Depletion Approach is Feasible for sOMV Vaccine Production

**Figure 4:**
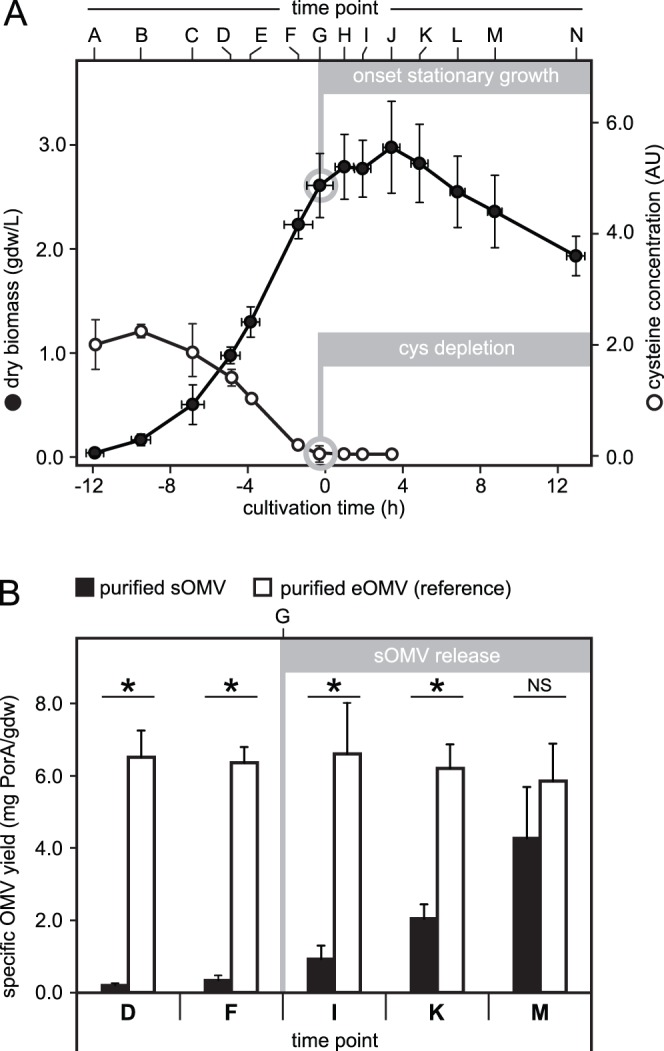
Implications for vaccine development. A novel approach for the production of sOMV vaccine against N. meningitidis serogroup B is explored by utilizing the effect of cysteine depletion. (A) Biomass concentration (closed circles) is monitored in bioreactor cultivations (time points A to N). Time point G marks onset of stationary growth, caused by depletion of cysteine (open circles). (B) Yield of purified sOMV vaccine (black bars) is compared with eOMV reference vaccine, which uses detergent-free biomass extraction to improve yield (white bars). Several time points before (D, F) and after (I, K, M) cysteine depletion are included. After cysteine depletion, sOMV yield increases gradually to quantities that are comparable to the eOMV reference (no significant difference at time point M). Significant yield differences are indicated with asterikses (p<0.05). ‘NS’ indicates a non-significant difference.

The shake flask repletion experiment measured sOMV yield directly in the supernatant without taking purification losses or vaccine quality into account. To assess whether the cysteine depletion approach was feasible for vaccine production, larger samples (250–400 mL) were taken from a bioreactor system to purify eOMV (reference vaccine) and sOMV (experimental vaccine) in parallel, according to the protocol in Figure S1. Purifications were done at several time points before and after cysteine depletion. The cultivations were monitored to measure biomass concentration and cysteine (time points A to N). As observed in shake flasks, biomass growth during the exponential phase was reproducible for all 6 replicate bioreactor cultivations (R^2^ = 0.975) and growth arrest ocurred upon cysteine depletion (time point G; Figure 4A).

**Figure 5:**
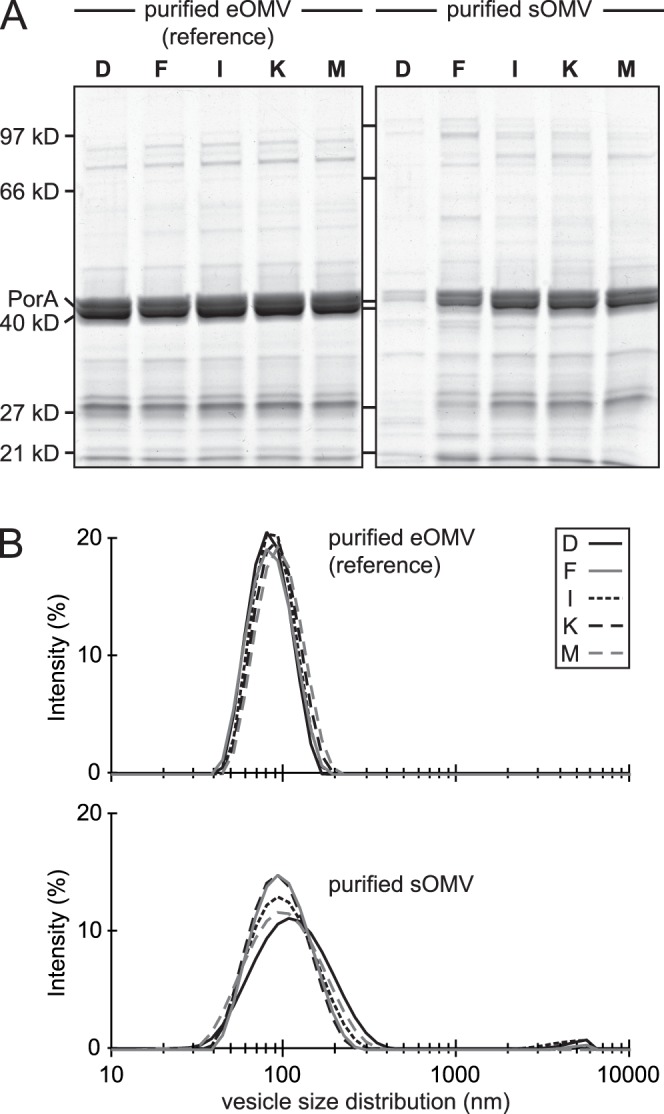
Quality of sOMV and eOMV vaccines. In addition to yield, quality of the sOMV and eOMV vaccines is compared. It was previously demonstrated that both vaccine types provide low toxicity and high functional immunogenicity in mice [11]. (A) Protein composition of eOMV reference vaccines is comparable to sOMV vaccines after cysteine depletion (time points I, K, M). Each lane contains 4 µg total protein, except sOMV at time points D and F (low protein concentration due to a low yield; maximal sample volume is loaded). PorA antigen (∼41 kD) has a major contribution to total protein content (>60%) in all vaccines. (B) Dynamic light scattering analysis reveals that sOMV vaccines have a slightly broader size distribution and minor aggregation compared to the eOMV references, indicating that the purification procedure is not yet fully consistent. X-axis represents vesicle size distribution (nm).

Purification of eOMV and sOMV vaccines was performed at time points D, F (before) and I, K, M (after cysteine depletion). Yield of purified eOMV (reference) correlated to the amount of biomass that was used, resulting in a constant amount of 6.3±0.3 mg PorA antigen/gdw regardless nutrient availability (Figure 4B). Yield of purified sOMV however depended on cysteine availability. Before depletion the yield was just above detection limit (0.2±0.1 and 0.3±0.1 mg PorA/gdw, respectively). Cysteine depletion then triggered sOMV release resulting in a cumulative increase during time point I (0.9±0.4 mg PorA/gdw), time point K (2.0±0.4 mg/gdw) and time point M (4.3±1.4 mg/gdw). The reference yield (eOMV) was significantly higher than sOMV at most time points (D, F, I and K; p<0.001) but no significant difference was found at time point M, indicating that the cysteine depletion approach can generate sufficient sOMV for feasible vaccine production. Protein composition and vesicle size distribution were measured to assess vaccine quality. Purified eOMV had a comparable overall protein compositon at all time points, with a high PorA antigen content (64±3% of total protein; Figure 5A). Purified sOMV were comparable to eOMV at time points F, I, K and M (PorA content 61±5%), but time point D had a low and variable purity (43±23%) caused by a yield that was below the threshold for reliable analysis. Vesicle size averages of eOMV (reference) and sOMV samples were comparable (83±5 and 97±9 nm respectively). The sOMV samples however had a slightly broader size distribution and contained minor particle peaks at ∼5 µm, indicating that the purification procedure may require improvements (Figure 5B).

## Discussion

This study with *Neisseria meningitidis* demonstrates that cysteine depletion can trigger growth arrest and the release of outer membrane vesicles (OMV) in sufficient quantities for vaccine production. The vesicles are released spontaneously (sOMV) and are as similar to *in vivo* vesicles as possible with a production system for human vaccines [5]. Therefore the obtained results may be relevant for the *in vivo* vesiculation and pathogenesis of *N. meningitidis*. To our knowledge, external stimuli that trigger sOMV release were not previously described for *N. meningitidis* or for cysteine as the limiting nutrient. Cysteine was found to be the growth-limiting component of human serum when supplemented to a chemically defined medium [42]. In serum-free media, some strains had an absolute cysteine requirement but others were able to grow on a variety of sulfur sources after adaptation [43], [44]. The vaccine production strain in this study is unable to adapt its sulfur metabolism after cysteine depletion, resulting in stationary growth despite a genetic ability to use alternate sulfur sources from the medium (Figure 1). The strain has specific mutations to express multiple protective PorA antigens, attenuate LPS toxicity and improve OMV yield, which are required for detergent-free vaccine production [11]. These mutations however may cause an inability to adapt to cysteine depleted conditions, since previous work with a strain that resembled the H44/76 parent strain more closely did not reveal an absolute cysteine requirement for growth [45].

Nutrient depletion was not previously associated with increased sOMV release by *N. meningitidis*, but specific genetic modifications are known to have a comparable outcome. The strain in this study has a disrupted *rmpM* gene to prevent anchoring of the outer membrane to the peptidoglycan layer with an OmpA-like domain [11], [46], [47]. The *gna33* mutation increases vesiculation through an unknown mechanism [12]. External stimuli for vesiculation have been identified in *E. coli*, including heat shock, lysine depletion or conditions that activate the σ^E^ envelope stress response pathway [31], [32], [33], [34]. In agreement with these observations, this study identified an external stress stimulus for *N. meningitidis* vesiculation. It remains unknown to what extent the current results are restricted to cysteine as the depleted nutrient or to this specific *N. meningitidis* strain.

Transcriptome analysis was performed to elucidate the intracellular effects of cysteine depletion in more detail. The transcriptome profile resembles studies with *N. meningitidis* that investigated stress [35], [36] and virulence stimuli [37], [38], [39] (Figure 2). Functional annotation revealed several pathways that are directly related to cysteine depletion or a decreased growth rate. The remaining pathways are related to oxidative stress, with a central role for iron-sulfur protein biogenesis. Iron-sulfur proteins are highly conserved in prokaryotes and eukaryotes. Mutants with an impaired biogenesis accumulate free intracellular iron and produce reactive oxygen species (ROS), which causes oxidative stress [48], [49]. In addition, iron-sulfur protein biogenesis requires sulfur acquisition and storage protein for free iron [50], [51]. These aspects are all represented by genes that are upregulated after cysteine depletion, including iron-sulfur cluster assembly (*erpA*, *nifU*), cysteine synthase/desulfurase (*cysK*, *iscS*), storage of Fe^2+^ ions (*bfrA*), ROS scavenging (*sodB*, *sodC*) and several reductases. Therefore it is hypothesized that impaired iron-sulfur protein biogenesis also occurs in *N. meningitidis* after cysteine depletion, causing increased intracellular iron levels and oxidative stress.

Recent work by our group showed that sOMV release by *N. meningitidis* increased significantly during the stationary growth phase, but the mechanism was not fully understood [23]. By comparing cysteine repleted with depleted conditions, this study demonstrates that cysteine depletion is the trigger for onset of stationary growth and increased sOMV release (Figure 3). It also shows that growth repression with oxidative stress, the hypothesised outcome of cysteine depletion, mimics this effect temporarily. Release of sOMV increases directly after each peroxide addition but then stabilizes, while cysteine depletion results in sustained vesicle release. Also, the peroxide dose range is narrow and critical for bacterial viability (data not shown). Despite these limitations, the results confirm involvement of oxidative stress as an intracellular signal for sOMV release and support the transcriptome data. *N. meningitidis* encounters oxidative stress *in vivo*, when phagocytes use ‘oxidative burst’ to eliminate the invading pathogen [52], [53]. In such conditions vesiculation may provide decoys for phagocytosis and enhance bacterial survival.

This study provides a novel approach for the production of sOMV vaccine against *N. meningitidis* serogroup B, in which onset of stationary growth and vesiculation is determined by the cysteine concentration of the growth medium. The use of a synthetic medium with a single growth-limiting component greatly improves process control. Other media for *N. meningitidis* have unidentified limiting nutrients and contain undefined components like casamino acids, resulting in less predictable growth [10], [17], . Purification of sOMV does not require concentration, resuspension or extraction of the bacterial biomass (Figure S1). These steps are labour intensive and complicate processing, especially when translated to large-scale processes. Therefore sOMV purification is the preferred approach for process development. To assess feasibility, yield and quality of sOMV vaccines were compared with eOMV references at several time points before and after cysteine depletion. In agreement with the initial observations (Figure 3), sOMV release is triggered after cysteine depletion and then increases over time. Final yield is comparable to the eOMV reference, indicating that cysteine depletion can also provide a feasible yield (Figure 4; ±1500 human doses sOMV vaccine per L cultivation). The sOMV have a slightly broader size distribution, indicating that the purification procedure is not yet fully consistent. This procedure may improve by replacing the centrifugal ultrafiltration units with more robust and scalable equipment like tangential flow ultrafiltration [56].

In conclusion, this study shows that cysteine depletion can trigger growth arrest and outer membrane vesicle release by *N. meningitidis*. Transcriptome data suggests a relation between cysteine depletion and impaired iron-sulfur protein assembly, resulting in oxidative stress. This hypothesis is substantiated by showing that induced oxidative stress during cysteine-rich growth also triggers vesiculation. Cysteine depletion improves the production of a vaccine against serogroup B meningococcal disease, resulting in substantial amounts of sOMV. Since the sOMV are more similar to vesicles from natural infection, cysteine-dependent vesiculation is likely to be relevant for the *in vivo* pathogenesis of *N. meningitidis*.

## Materials and Methods

### Bacterial Strain and Shake Flask Cultivations

The *N. meningitidis* vaccine strain that was used is a recombinant variant of serogroup B isolate H44/76 [57], combining one wild-type and two recombinant PorA antigens (trivalent PorA; subtypes P1.7,16; P1.5–1,2–2 and P1.19,15–1) with a non-functional *porB* gene [58]. The *cps* locus was deleted, resulting in a non-encapsulated phenotype with *galE*-truncated LPS. Additional deletions in *lpxL1* and *rmpM* genes attenuate LPS toxicity and improve yield [11]. All cultures were grown in chemically defined growth medium [59]. For shake flask cultivations, 150 mL pre-culture was inoculated with 10 mL working seedlot (cells at OD_590_ = 1.5±0.1; stored at −135°C with glycerol) and incubated in 500 mL erlenmeyer shake flasks (Nalgene, Rochester NY, U.S.A.) at 35°C, 200 rpm. At an OD_590_ of 1.5±0.3, 10 mL portions from the pre-culture shake flask were used to inoculate several secondary shake flasks, containing media with different arginine and cysteine concentrations (for Figure 1). Alternatively, secondary shake flasks with normal medium were grown to an OD_590_ of 2.5±0.3 for repletion experiments (for Figure 3). Shake flasks for Figure 3 were pooled and centrifuged in 150 mL portions (20 min.; 3000×g; 4°C). Pellets were resuspended, washed in 75 mL medium (with/without cysteine) and re-centrifuged. The depletion/repletion experiments started after resuspending the pellets in 150 mL medium with/without cysteine. To induce oxidative stress, 150 µM peroxide was added to each shake flask (triplicates) directly after depletion/repletion (t = 0). Growth was then monitored with hourly OD_590_ measurements. If the average optical density increased >10% between 2 measurements, 50 µM peroxide was added to repress growth. This was necessary at t = 2.5 and 5.5 hours after depletion/repletion (arrows in Figure 3).

### Bioreactor Cultivations

Transcriptome analysis (Figure 2) and OMV production (Figure 4) was performed on samples from a fully instrumented, 5 L benchtop bioreactor (Applikon, Schiedam, The Netherlands) with 6-blade Rushton impeller for mixing and gas dispersion and initial working volume of 3.5 L. Controller set points were 35.0±0.5°C (temperature), 30±5% (dissolved oxygen concentration), 7.2±0.1 (pH), 300–750 rpm (stirrer speed). The bioreactor was inoculated with 150 mL pre-culture at OD_590_ = 1.5±0.3. The bioreactor was connected to an ADI-1040 control system (Applikon), operated with BCSV software (Compex, Gent, Belgium). After inoculation, dissolved oxygen concentration was first controlled by gradually increasing stirrer speed to 750 rpm. Then the fraction of oxygen in the headspace increased gradually while maintaining a constant total gas flow of 1.0 L/min. Samples for nutrient analysis were sterile filtered (0.22 µm) and stored at 4°C.

### Transcriptome Analysis

Transcriptome analysis was performed as described previously [60], [61]. RNA were analysed with a full-genome *N. meningitidis* MC58 microarray [41], which covered 93% of predicted ORFs (Operon, Köln, Germany). Oligonucleotides were spotted in triplicate on UltraGAPS II coated slides (Corning, Corning NY, USA). The transcriptome profile was preserved by mixing 1 volume of bacterial culture (corresonding to 2.5 mL at OD_590_ = 1) with 2 volumes of RNA-later solution (Ambion, Paisley, United Kingdom), then concentrated with centrifugation (20 min.; 3000×g; 4°C) and stored at −80°C until RNA extraction. Bacterial pellets were thawed and pre-treated in Tris-EDTA buffer with 0.5 mg/mL lysozyme (Sigma–Aldrich, Zwijndrecht, The Netherlands) prior to RNA extraction according to manufacturer’s protocol (SV Total RNA isolation kit; Promega, Fitchburg WI, USA). Nucleic acid concentration was adjusted with ethanol precipitation and spectral analysis was used to determine purity and concentration. RNA integrity was measured with the Bioanalyzer RNA6000 Nano assay (Agilent Technologies, Santa Clara CA, USA), according to the manufacturers’ protocol. RNA integrity number (RIN) scores were used to assess RNA integrity (score >8.0 required for inclusion) [62]. Total RNA from triplicate samples at each time point were reverse transcribed to cDNA and labelled with Cy3 dye using the Chipshot Indirect Labeling and Clean-Up kit (Promega), according to the manufacturer’s protocol. Common reference samples, containing equal amounts of total RNA from all experimental samples, were labelled with Cy5. The labelled and purified cDNA samples were pooled in Cy3/Cy5 pairs. Hybridization buffer was added to a final concentration of 25% formamide, 56 SSC and 0.1% SDS. Samples were applied to the microarray slides and incubated in a hybridization chamber (16–20 h; 42°C; dark). Differential gene expression was calculated through comparison with the common reference. Microarrays were scanned with a ScanArray Express microarray scanner (Perkin Elmer, Waltham MA, USA) and median fluorescence intensities were quantified for each spot using ArrayVision software (Imaging Research). The data was natural-log transformed, quantile normalized and values of replicate spots were averaged. These data processing steps were done with the statistical software R, using an in-house developed script. P-values were calculated with one-way ANOVA statistical analysis. Significantly regulated genes were selected with a False Discovery Rate (FDR) of <10% to adjust p-values for multiple testing. Fold ratio (FR) values were expressed as the natural log of the normalized signal difference between the two groups. To further select for biologically relevant effects, a FR threshold of >2.0 (untransformed value) was applied to obtain the final results. Gene annotations were obtained from Uniprot Knowledge Base (www.uniprot.org; *N. meningitidis* MC58; version July 2011). Principal component analysis (PCA) and clustering of differentially expressed genes in expression groups was performed with Genemaths XT software (Applied Maths, Sint-Martens-Latem, Belgium). Overall gene expression differences between samples were assessed by calculating the Euclidian distance between gene expression values over the entire set of genes (this corresponds to the distance in a PCA plot). Functional annotations of cysteine regulated genes were retrieved from the Gene Ontology database, using corresponding Uniprot protein IDs (www.geneontology.org; version February 2012). Enrichment for a specific Gene Ontology was assessed by comparing the number of hits in the cysteine dataset with the number of hits in the MC58 genome. Enrichment p-values were obtained by calculating the binomial distrbution probability. The binomial distribution probability was also used to calculate a p-value for the overlap between gene lists, allowing comparison of the cysteine dataset with relevant literature.

### OMV Purification and Characterization

This study used small-scale, detergent-free OMV purifications as described previously [11]. Cultivation samples (250–400 mL) were taken from the bioreactor and purified. The samples were split in supernatant (sOMV) and pellet (eOMV) with centrifugation (20 min.; 3000×g; 4°C). To purify sOMV, supernatant was sterile filtered (0.22 µm) and concentrated with ultrafiltration (UF), using centrifugal units with 100 kD cutoff (Centricon 70-plus Ultracell, Millipore, Billerica MA, USA). UF units were washed with saline, filled with 70 mL supernatant and concentrated to 10–15 mL by centrifugation. The retentate was re-diluted to 70 mL with fresh supernatant and the cycle was repeated until the full sample was processed (centrifugation at 1500×g; 4°C). Final retentate was diluted to 70 mL with storage buffer and concentrated to 10 mL (first wash step). The sOMV in the retentate were then pelleted with ultracentrifugation (120 min.; 125000×g; 4°C) and resuspended in a suitable volume of storage buffer (second wash step). To purify eOMV, the bacterial pellet was resuspended in 7.5 volumes (mL/g wet weight) of EDTA buffer (100 mM Tris-HCl pH8.6 with 10 mM EDTA) and incubated (30 min. ambient temperature). Cells were discarded by semi high-speed centrifugation (75 min.; 20000×g; 4°C). Ultracentrifugation was used to pellet eOMV in the supernatant (120 min.; 125000×g; 4°C) and the pellet was resuspended in a suitable volume of storage buffer. Final total protein concentration was 1.0±0.5 mg/mL for all OMV samples. Total protein concentration, PorA quantity and vesicle size distribution of OMV samples were performed as described previously [11], [63]. Briefly, total protein concentration was measured with the Lowry protein assay. Peterson’s modification was used to reduce the effect of interfering substances. PorA antigen content was determined by SDS gel electrophoresis, followed by total protein staining and quantification of the 40–44 kD bands (PorA). Gels were stained with Novex Colloidal Blue (Invitrogen, Breda, The Netherlands) and PorA was quantified as a percentage of total protein using TL100 1D gel analysis software (Totallab, Newcastle upon Tyne, U.K.). Vesicle size distribution was measured with dynamic light scattering (DLS) at 25°C with a Malvern 4700 system. Homogeneity of the vesicle size distribution was reflected in the polydispersity index (PdI), which ranges between 0.0 (fully homogeneous size distribution) and 1.0 (random size distribution).

### Quantification of Nutrients and sOMV Release

Cysteine (after reaction with bromoacetic acid and reduction using tris(2-ethylcarboxy)phosphine (TCEP)) and arginine were quantified by HPLC after derivatisation with orthophtalic anhydride (OPA). Release of sOMV in the culture supernatant was monitored by using the fluorescent signal of a phospholipid-specific probe (SynaptoRed C2, Biotium, Hayward, CA, USA; ex500, em650). An aqueous solution of SynaptoRed C2 (0.05 mM, 50 µL) was mixed with 50 µL of sterile filtered culture supernatant or OMV standard with a known concentration. Fluorescence of the resulting mixture was recorded in black microtiter plates using a fluorometer (Synergy MX microplate reader; Biotek, Bad Friedrichshall, Germany). A calibration curve was constructed from the responses of the standards (dilutions of purified eOMV standard corresponding to 0–10 mg/L PorA antigen). Concentration of sOMV in culture supernatant samples was calculated from this calibration curve.

## Supporting Information

Figure S1 **Detergent-free OMV purification.** There are two different detergent-free OMV purification types; sOMV (spontaneously released OMV) are purified from the culture supernatant without additional treatments to enhance vesicle release, while eOMV (extracted OMV) are obtained after detergent-free extraction of the bacterial biomass with a chelating agent (EDTA). Therefore both OMV types can be purified in parallel from a single cultivation. This study uses eOMV as a reference for high yield and quality [23]. Cultivation harvest was split in supernatant and bacterial pellet with low speed centrifugation. The supernatant was sterilized with filtration, then sOMV were concentrated with ultrafiltration and pelleted with ultracentrifugation. Purified sOMV were resuspended in a suitable volume of storage buffer. The bacterial pellet was resuspended in EDTA extraction buffer to release eOMV. Cells were incubated and discarded with semi high-speed centrifugation. Ultracentrifugation was used to concentrate eOMV and the pellet was resuspended in a suitable volume of storage buffer. Purification of eOMV requires several complicated steps in terms of handling (concentration, resuspension and extraction of the bacterial biomass). Therefore sOMV purification is the preferred approach for process development.
(PDF)

Figure S2 **Nutrient monitoring identifies the growth-limiting medium component.** Media with different combinations of cysteine (cys) and arginine (arg) are tested to determine the growth-limiting component. Upper plots represent nutrient concentrations in media with normal cys, while the lower plots represent media with low cys. Media with normal or low arg are indicated with circles or triangles, respectively. Y-axes represent cys concentration (plots on the left) or arg concentration (plots on the right), The X-axis represents cultivation time, with t = 0 as the expected time point of growth arrest (confirmed for control medium with normal amounts of cys and arg; upper left plot, circles). Grey vertical lines indicate the observed growth arrest time point (obtained from Figure 1). The results demonstrate that growth arrest depends on cys availability and is independent of arg.
(PDF)

Figure S3 **Detailed transcriptome analysis.** (A) Principal component analysis was performed on the full transcriptome to asses reproducibility of replicates and identify sources of variation in the dataset (principal components, or PCs). The analysis shows that PC1 and PC2 explain 70% and 7% of total variation in the dataset, respectively, while the remaining PCs explained <5% each. PC1 represents cysteine availability because all samples before depletion (t = −2.5 h (white circles), t = −1.2 h (grey circles) and t = −0.4 h (black circles)) have a high score, while depleted samples (t = +1.4 h (white triangles) and t = +3.5 h (black triangles)) have a low score. This indicates that the experimental treatment accounts for the majority of variation in the data. All biological replicates have comparable PC1 and PC2 coordinates, indicating high reproducibility. (B) Significant effects in the dataset were initially selected with a False Discovery Rate threshold (FDR <0.10). To refine for biologically relevant effects, a Fold Ratio filter was used (FR >2.0). Clustering of the resulting 149 cysteine regulated genes reveals 3 expression groups: downregulated after cysteine depletion (48 genes), upregulated after depletion (90 genes) and 11 genes with a variable expression pattern. For further interpretation of the results, functional properties of the cysteine regulated genes were assessed. This resulted in 36 enriched Gene Ontologies (p<0.05) from 11 functional groups (Table S1).
(PDF)

Table S1 **Cysteine regulated genes with functional annotation.** (A) List of all genes that are differentially expressed after cysteine depletion. Each entry contains accession number, gene name, gene symbol, expression data (pattern and statistics) and details of the functional annotation (enriched Gene Ontology numbers and corresponding functional groups). (B) Overview of enriched Gene Ontologies in the cysteine depletion dataset. Each entry contains Gene Ontology ID, name and corresponding functional group. The statistics section shows the number of hits for each Gene Ontology in the cysteine depletion dataset and the total number of hits in the MC58 genome. Based on both counts, an enrichment p-value is calculated by using the binomial distribution probability. Only Gene Ontologies with significant p-values (<0.05) are included in the list.
(PDF)
