# Supplementary figures and images for: Cysteine Depletion Causes Oxidative Stress and Triggers Outer Membrane Vesicle Release by Neisseria meningitidis; Implications for Vaccine Development
Source: PLoS One. 2013 Jan 23;8(1):e54314. doi: 10.1371/journal.pone.0054314 (PMC3553081; doi:10.1371/journal.pone.0054314)

**eOMV purification**  
(extraction with EDTA)

**sOMV purification**  
(spontaneous release)

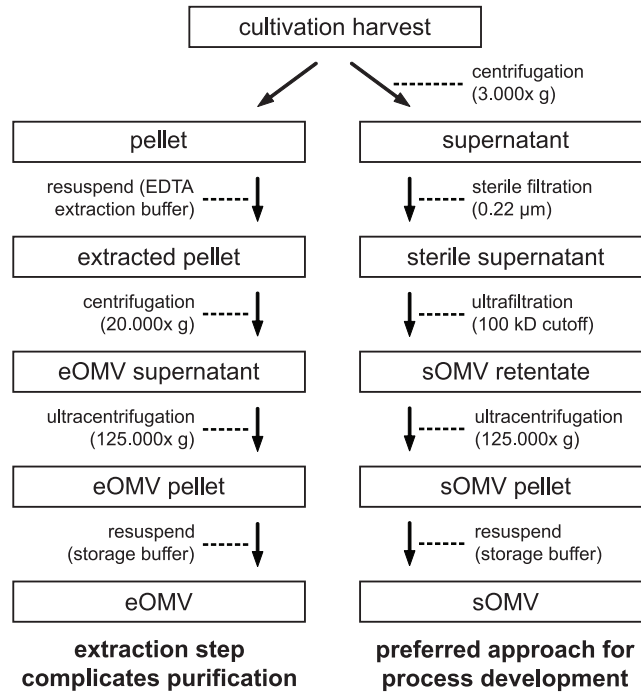

Supplement: Figure S1 — Detergent-free OMV purification. There are two different detergent-free OMV purification types; sOMV (spontaneously released OMV) are purified from the culture supernatant without additional treatments to enhance vesicle release, while eOMV (extracted OMV) are obtained after detergent-free extraction of the bacterial biomass with a chelating agent (EDTA). Therefore both OMV types can be purified in parallel from a single cultivation. This study uses eOMV as a reference for high yield and quality [23]. Cultivation harvest was split in supernatant and bacterial pellet with low speed centrifugation. The supernatant was sterilized with filtration, then sOMV were concentrated with ultrafiltration and pelleted with ultracentrifugation. Purified sOMV were resuspended in a suitable volume of storage buffer. The bacterial pellet was resuspended in EDTA extraction buffer to release eOMV. Cells were incubated and discarded with semi high-speed centrifugation. Ultracentrifugation was used to concentrate eOMV and the pellet was resuspended in a suitable volume of storage buffer. Purification of eOMV requires several complicated steps in terms of handling (concentration, resuspension and extraction of the bacterial biomass). Therefore sOMV purification is the preferred approach for process development. (PDF) [file pone.0054314.s001.pdf]

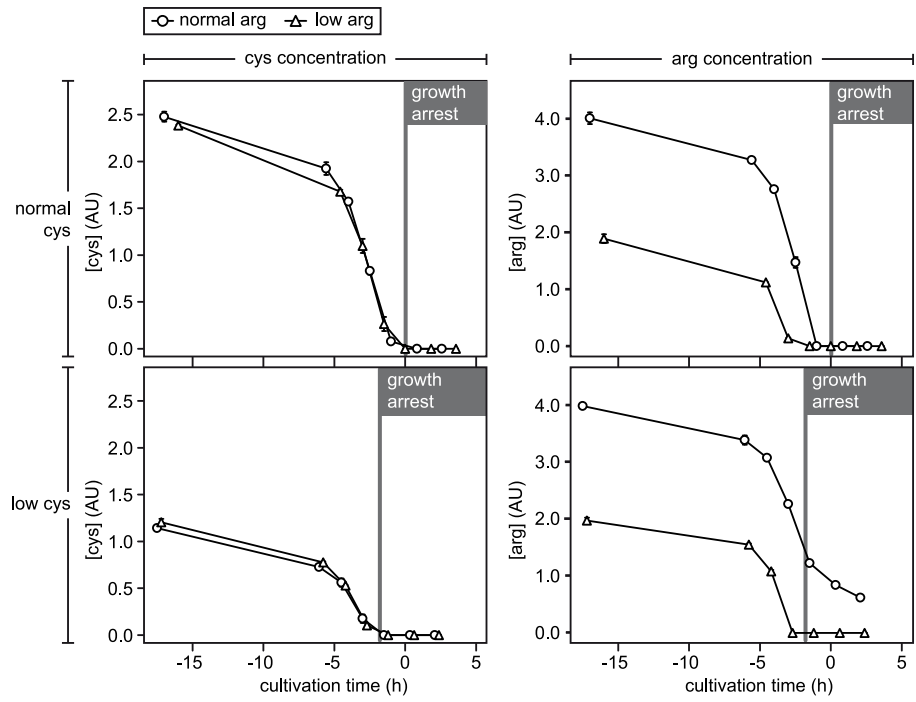

Supplement: Figure S2 — Nutrient monitoring identifies the growth-limiting medium component. Media with different combinations of cysteine (cys) and arginine (arg) are tested to determine the growth-limiting component. Upper plots represent nutrient concentrations in media with normal cys, while the lower plots represent media with low cys. Media with normal or low arg are indicated with circles or triangles, respectively. Y-axes represent cys concentration (plots on the left) or arg concentration (plots on the right), The X-axis represents cultivation time, with t = 0 as the expected time point of growth arrest (confirmed for control medium with normal amounts of cys and arg; upper left plot, circles). Grey vertical lines indicate the observed growth arrest time point (obtained from Figure 1). The results demonstrate that growth arrest depends on cys availability and is independent of arg. (PDF) [file pone.0054314.s002.pdf]

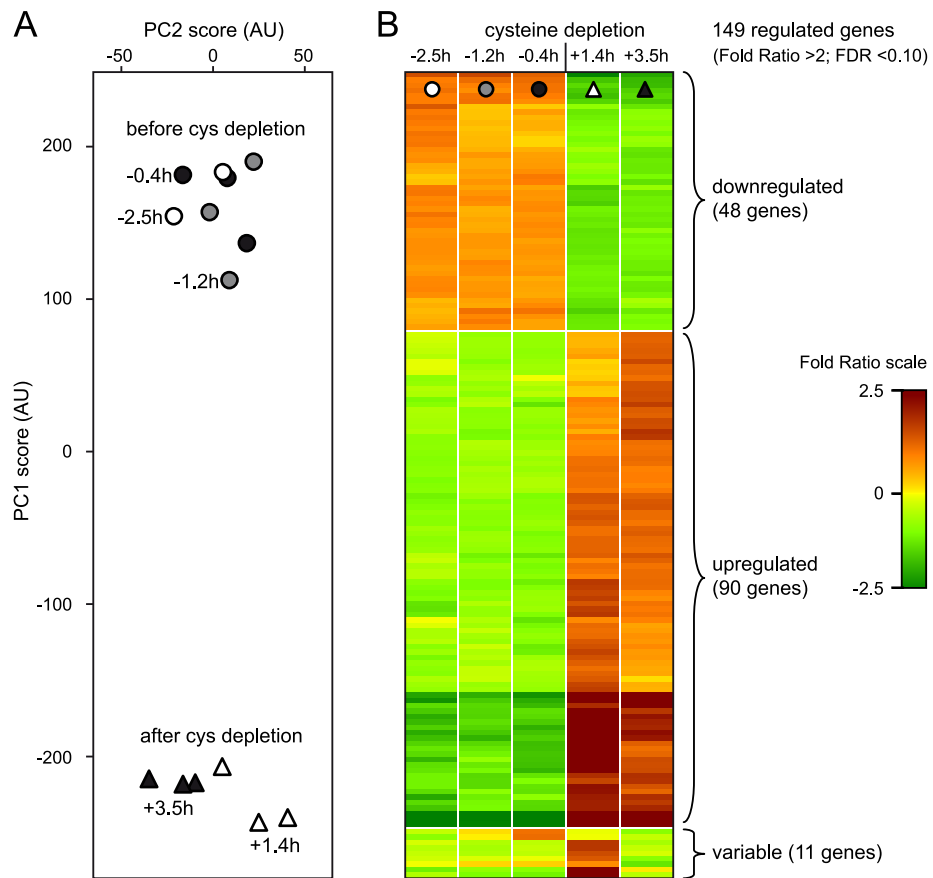

Supplement: Figure S3 — Detailed transcriptome analysis. (A) Principal component analysis was performed on the full transcriptome to asses reproducibility of replicates and identify sources of variation in the dataset (principal components, or PCs). The analysis shows that PC1 and PC2 explain 70% and 7% of total variation in the dataset, respectively, while the remaining PCs explained <5% each. PC1 represents cysteine availability because all samples before depletion (t = −2.5 h (white circles), t = −1.2 h (grey circles) and t = −0.4 h (black circles)) have a high score, while depleted samples (t = +1.4 h (white triangles) and t = +3.5 h (black triangles)) have a low score. This indicates that the experimental treatment accounts for the majority of variation in the data. All biological replicates have comparable PC1 and PC2 coordinates, indicating high reproducibility. (B) Significant effects in the dataset were initially selected with a False Discovery Rate threshold (FDR <0.10). To refine for biologically relevant effects, a Fold Ratio filter was used (FR >2.0). Clustering of the resulting 149 cysteine regulated genes reveals 3 expression groups: downregulated after cysteine depletion (48 genes), upregulated after depletion (90 genes) and 11 genes with a variable expression pattern. For further interpretation of the results, functional properties of the cysteine regulated genes were assessed. This resulted in 36 enriched Gene Ontologies (p<0.05) from 11 functional groups (Table S1). (PDF) [file pone.0054314.s003.pdf]
