# Supplementary material for: Cysteine Depletion Causes Oxidative Stress and Triggers Outer Membrane Vesicle Release by Neisseria meningitidis; Implications for Vaccine Development
Source: PLoS One. 2013 Jan 23;8(1):e54314. doi: 10.1371/journal.pone.0054314 (PMC3553081; doi:10.1371/journal.pone.0054314)
Supplement: Table S1 — Cysteine regulated genes with functional annotation. (A) List of all genes that are differentially expressed after cysteine depletion. Each entry contains accession number, gene name, gene symbol, expression data (pattern and statistics) and details of the functional annotation (enriched Gene Ontology numbers and corresponding functional groups). (B) Overview of enriched Gene Ontologies in the cysteine depletion dataset. Each entry contains Gene Ontology ID, name and corresponding functional group. The statistics section shows the number of hits for each Gene Ontology in the cysteine depletion dataset and the total number of hits in the MC58 genome. Based on both counts, an enrichment p-value is calculated by using the binomial distribution probability. Only Gene Ontologies with significant p-values (<0.05) are included in the list. (PDF) [file pone.0054314.s004.pdf]

Supplementary Table S1A - Cysteine regulated genes (page 1/3)

Gene information				Transcriptome data			Functional annotation	
Gene ID	Uniprot ID	Gene product name	Symbol	Expression	FoldRatio (>2)	FDR (<0.1)	Enriched Gene Ontology (p<0.05)	functional group
NMB0131	P0A0X1	50S ribosomal protein L7/L12	rplL	downregulated	2.17	0.0161	0003735; 0005622; 0005840; 0006412; 0030529	translation; other functions
NMB0142	P60444	50S ribosomal protein L3	rplC	downregulated	2.27	0.0001	0003735; 0005622; 0005840; 0006412; 0030529	translation; other functions
NMB0144	Q9K1I6	50S ribosomal protein L23	rplW	downregulated	2.09	0.0005	0003735; 0005622; 0005840; 0006412; 0030529	translation; other functions
NMB0148	P66551	30S ribosomal protein S3	rpsC	downregulated	2.09	0.0001	0003735; 0005622; 0005840; 0006412; 0030529	translation; other functions
NMB0149	Q7DDT4	50S ribosomal protein L16	rplP	downregulated	2.06	0.0001	0003735; 0005622; 0005840; 0006412; 0030529	translation; other functions
NMB0150	P66169	50S ribosomal protein L29	rpmC	downregulated	2.17	0.0003	0003735; 0005622; 0005840; 0006412; 0030529	translation; other functions
NMB0151	Q7DDT3	30S ribosomal protein S17	rpsQ	downregulated	2.14	0.0004	0003735; 0005622; 0005840; 0006412; 0030529	translation; other functions
NMB0265	Q9K1A2	Holliday junction ATP-dependent DNA helicase ruvA	ruvA	downregulated	2.11	0.0001	not found	not found
NMB0266	Q9K1A1	Putative uncharacterized protein	-	downregulated	2.11	0.0039	not found	not found
NMB0281	Q9K186	Peptidyl-prolyl cis-trans isomerase (EC 5.2.1.8)	-	downregulated	2.15	0.0036	not found	not found
NMB0320	Q9K158	Putative uncharacterized protein	-	downregulated	2.24	0.0023	not found	not found
NMB0324	P66130	50S ribosomal protein L27	rpmA	downregulated	2.29	0.0397	0003735; 0005622; 0005840; 0006412; 0030529	translation; other functions
NMB0378	Q7DDQ8	Putative phosphate permease	-	downregulated	2.12	0.0014	not found	not found
NMB0462	Q9K0U8	Spermidine/putrescine ABC transporter	potD1	downregulated	2.01	0.0003	0005215; 0006810	membrane transport
NMB0565	Q9K0M7	Na(+)-translocating NADH-quinone reductase subunit E	nqrE	downregulated	2.33	0.0001	0006810; 0006811; 0006814; 0009276; 0016491; 0016655; 0022904; 0055114	membrane transport; cell wall; redox
NMB0566	Q9K0M6	Na(+)-translocating NADH-quinone reductase subunit D	nqrD	downregulated	2.11	0.0001	0006810; 0006811; 0006814; 0016491; 0016655; 0055114	membrane transport; redox
NMB0567	Q9K0M5	Na(+)-translocating NADH-quinone reductase subunit C	nqrC	downregulated	2.47	0.0001	0006810; 0006811; 0006814; 0009276; 0010181; 0016491; 0016655; 0055114	membrane transport; cell wall; redox
NMB0568	Q9K0M4	Na(+)-translocating NADH-quinone reductase subunit B	nqrB	downregulated	2.14	0.0000	0006810; 0006811; 0006814; 0009276; 0010181; 0016491; 0016655; 0022904; 0055114	membrane transport; cell wall; redox
NMB0569	Q9K0M3	Na(+)-translocating NADH-quinone reductase subunit A	nqrA	downregulated	2.43	0.0000	0006810; 0006811; 0006814; 0016491; 0016655; 0055114	membrane transport; redox
NMB0590	Q9K0K4	tRNA (guanine-N(1)-)-methyltransferase (EC 2.1.1.31)	trmD	downregulated	2.13	0.0001	not found	not found
NMB0591	Q9K0K3	Ribosome maturation factor rimM	rimM	downregulated	2.14	0.0001	5840	translation
NMB0592	P66438	30S ribosomal protein S16	rpsP	downregulated	2.04	0.0000	0003735; 0005622; 0005840; 0006412; 0030529	translation; other functions
NMB0721	P65138	Translation initiation factor IF-3	infC	downregulated	2.54	0.0000	6412	translation
NMB0742	Q9K076	Putative uncharacterized protein	-	downregulated	2.18	0.0009	55114	redox
NMB0943	Q9JZQ3	5,10-methylenetetrahydrofolate reductase (EC 1.5.1.20)	metF	downregulated	2.92	0.0000	0008652; 0016491; 0055114	amino acid biosynthesis; redox
NMB0944	Q9JZQ2	homocysteine methyltransferase (EC 2.1.1.14)	metE	downregulated	2.76	0.0001	0008652; 0046872	amino acid biosynthesis; metal ion uptake
NMB0977	Q9JZN3	Putative modulator of drug activity B	-	downregulated	2.06	0.0203	0016491; 0055114	redox
NMB0986	Q9JZM5	Putative uncharacterized protein	-	downregulated	2.02	0.0025	not found	not found
NMB1277	Q9JZ61	Transporter, BCCT family	-	downregulated	2.18	0.0002	0005215; 0006810	membrane transport
NMB1285	Q9JZ53	Enolase (EC 4.2.1.11)	eno	downregulated	2.63	0.0001	46872	metal ion uptake
NMB1289	Q9JZ51	Putative type II restriction enzyme	-	downregulated	2.04	0.0061	not found	not found
NMB1290	Q7DDD1	Cytosine-specific methyltransferase (EC 2.1.1.37)	-	downregulated	2.18	0.0016	not found	not found
NMB1362	Q9JYZ3	Putative oxalate/formate antiporter	-	downregulated	2.67	0.0004	not found	not found
NMB1363	Q9JYZ2	Exodeoxyribonuclease 7 large subunit (EC 3.1.11.6)	xseA	downregulated	2.10	0.0008	not found	not found
NMB1426	Q7DDB9	Putative uncharacterized protein	-	downregulated	2.80	0.0025	not found	not found
NMB1427	Q9JYU5	Putative uncharacterized protein	-	downregulated	2.06	0.0011	not found	not found
NMB1430	Q9JYU3	Transcription elongation factor greA	greA	downregulated	2.14	0.0000	not found	not found
NMB1432	Q9JYU1	3-phosphoshikimate 1-carboxyvinyltransferase	aroA	downregulated	4.31	0.0000	8652	amino acid biosynthesis
NMB1568	Q9JYI7	Putative DNA polymerase holoenzyme chi subunit	-	downregulated	2.21	0.0004	not found	not found
NMB1647	Q9JYC8	Putative amino acid symporter	-	downregulated	2.05	0.0035	0005283; 0006814	membrane transport
NMB1857	Q9JXW6	Modulator of drug activity B	mdaB	downregulated	2.58	0.0130	0016491; 0055114	redox
NMB1904	P66251	50S ribosomal protein L34	rpmH	downregulated	2.24	0.0012	0003735; 0005622; 0005840; 0006412; 0030529	translation; other functions
NMB1920	Q9JXR2	GMP synthase [glutamine-hydrolyzing] (EC 6.3.5.2)	guaA	downregulated	2.15	0.0001	not found	not found
NMB1933	Q9JXQ3	ATP synthase epsilon chain	atpC	downregulated	2.62	0.0056	0006810; 0006811	membrane transport
NMB1975	Q9JXM3	Transporter	-	downregulated	2.09	0.0017	6810	membrane transport
NMB2056	P66642	30S ribosomal protein S9	rpsI	downregulated	2.09	0.0003	0003735; 0005622; 0005840; 0006412; 0030529	translation; other functions
NMB2129	Q9JXC1	Argininosuccinate synthase (EC 6.3.4.5)	argG	downregulated	2.31	0.0006	8652	amino acid biosynthesis
NMB2132	Q7DD37	Transferrin-binding protein-related protein	-	downregulated	2.20	0.0002	not found	not found
NMB0059	P63969	Chaperone protein DnaJ	dnaJ	upregulated	2.12	0.0106	0009408; 0046872	stress; metal ion uptake
NMB0092	Q9K1L9	Putative uncharacterized protein	-	upregulated	2.11	0.0000	not found	not found
NMB0093	Q9K1L8	Putative uncharacterized protein	-	upregulated	2.04	0.0001	not found	not found
NMB0177	Q9K1H4	Putative sodium/alanine symporter	-	upregulated	2.11	0.0099	0005283; 0006814	membrane transport
NMB0329	Q7DDR2	Type IV pilus assembly protein	pilF	upregulated	2.29	0.0008	0005622; 0006810	other functions; membrane transport
NMB0364	Q9K129	FrpC operon protein	-	upregulated	2.07	0.0005	not found	not found

continued (page 2/3)

Gene information				Transcriptome data			Functional annotation	
**Gene ID**	**Uniprot ID**	**Gene product name**	**Symbol**	**Expression**	**FoldRatio (>2)**	**FDR (<0.1)**	**Enriched Gene Ontology (p<0.05)**	**functional group**
NMB0393	Q9K106	Multidrug resistance protein	-	upregulated	2.57	0.0001	6810	membrane transport
NMB0501	Q9JRV6	Putative uncharacterized protein	-	upregulated	2.28	0.0003	not found	not found
NMB0509	Q9K0S0	Putative uncharacterized protein	-	upregulated	2.16	0.0017	not found	not found
NMB0555	Q9K0N3	Putative uncharacterized protein	-	upregulated	2.49	0.0120	not found	not found
NMB0557	Q7DDN1	Putative iron-sulfur cluster insertion protein erpA	erpA	upregulated	2.36	0.0085	0005198; 0016226; 0046872; 0051536	iron-sulfur cluster; metal ion uptake
NMB0584	Q9K0L0	FrpC operon protein	-	upregulated	2.06	0.0001	not found	not found
NMB0586	Q9K0K8	Putative adhesin	-	upregulated	4.43	0.0013	0006810; 0046872	membrane transport; metal ion uptake
NMB0604	Q9K0J3	Alcohol dehydrogenase, zinc-containing	-	upregulated	4.47	0.0000	0016491; 0046872; 0055114	redox; metal ion uptake
NMB0649	Q9K0F7	Putative uncharacterized protein	-	upregulated	2.08	0.0078	not found	not found
NMB0671	Q9K0D8	Malate oxidoreductase (NAD) (EC 1.1.1.38)	sfcA	upregulated	2.17	0.0033	0016491; 0046872; 0055114	redox; metal ion uptake
NMB0679	Q9K0D3	Acetyl-CoA carboxylase	accD	upregulated	2.17	0.0009	not found	not found
NMB0701	Q9K0B3	Putative uncharacterized protein	-	upregulated	2.84	0.0060	not found	not found
NMB0711	Q9K0A4	Putative uncharacterized protein	-	upregulated	2.96	0.0003	not found	not found
NMB0763	Q7DDL5	Cysteine synthase (CSase) (EC 2.5.1.47)	cysK	upregulated	5.49	0.0001	0008652; 0019344	amino acid biosynthesis; sulfur metabolism
NMB0787	Q9K035	Amino acid ABC transporter, periplasmic protein	-	upregulated	3.80	0.0007	0005215; 0006810	membrane transport
NMB0788	Q9K034	Amino acid ABC transporter, permease protein	-	upregulated	2.31	0.0009	0005215; 0006810	membrane transport
NMB0789	Q9K033	Amino acid ABC transporter, ATP-binding protein	-	upregulated	2.27	0.0132	not found	not found
NMB0814	Q9K013	ATP phosphoribosyltransferase regulatory subunit	hisZ	upregulated	2.09	0.0000	0006412; 0008652	translation; amino acid biosynthesis
NMB0815	Q9K012	Adenylosuccinate synthetase (AMPSase)	purA	upregulated	2.22	0.0000	46872	metal ion uptake
NMB0879	Q9JZW0	Sulfate/thiosulfate import ATP-binding protein CysA	cysA	upregulated	4.87	0.0006	0005215; 0006810; 0008272; 0015419	membrane transport; sulfur metabolism
NMB0880	Q9JZV9	Sulfate ABC transporter, permease protein	cysW	upregulated	2.63	0.0014	0005215; 0006810; 0008272	membrane transport; sulfur metabolism
NMB0884	Q9JZV6	Superoxide dismutase (EC 1.15.1.1)	sodB	upregulated	2.60	0.0001	0004784; 0006801; 0016491; 0046872; 0055114	redox; metal ion uptake
NMB0919	Q9JZS2	Putative IS1106 transposase	-	upregulated	2.36	0.0025	not found	not found
NMB0941	P66295	50S ribosomal protein L36 2	rpmJ2	upregulated	15.54	0.0003	0003735; 0005622; 0005840; 0006412; 0030529	translation; other functions
NMB0942	Q9JZQ4	50S ribosomal protein L31 type B	rpmE2	upregulated	32.11	0.0000	0003735; 0005622; 0005840; 0006412; 0030529	translation; other functions
NMB0946	Q7DDK4	Peroxiredoxin 2 family protein/glutaredoxin	-	upregulated	2.20	0.0043	0016491; 0055114	redox
NMB0951	Q7DDK2	Succinate dehydrogenase, iron-sulfur protein (EC 1.3.99.1)	sdhB	upregulated	2.85	0.0001	0006099; 0016491; 0051536; 0055114	energy metabolism; redox; iron-sulfur cluster
NMB0952	Q7DDK1	Putative uncharacterized protein	-	upregulated	2.36	0.0002	not found	not found
NMB0953	Q9JZP7	Putative uncharacterized protein	-	upregulated	2.15	0.0012	not found	not found
NMB0954	Q7DDK0	Citrate synthase (EC 2.3.3.1)	gltA	upregulated	2.46	0.0003	0004108; 0006099; 0044262; 0046912	energy metabolism; other functions
NMB0964	Q9JZN9	Probable tonB-dependent receptor NMB0964	-	upregulated	2.50	0.0103	0005215; 0006810	membrane transport
NMB0966	Q9JZN7	Para-aminobenzoate synthase	pabA	upregulated	2.73	0.0000	not found	not found
NMB0991	Q9JZM0	IS1106 transposase	-	upregulated	2.20	0.0063	not found	not found
NMB0994	Q9JZL9	Acyl-CoA dehydrogenase family protein	-	upregulated	3.51	0.0019	55114	redox
NMB0995	Q9JZL8	Macrophage infectivity potentiator-related protein	-	upregulated	7.20	0.0003	0004601; 0016209; 0016491; 0055114	redox
NMB1017	Q9JZK1	Sulfate ABC transporter, periplasmic sulfate-binding protein	sbp	upregulated	2.94	0.0029	0005215; 0006810; 0008272; 0015419	membrane transport; sulfur metabolism
NMB1054	not available	-	-	upregulated	2.16	0.0080	not found	not found
NMB1055	P56990	Serine hydroxymethyltransferase (SHMT)	glyA	upregulated	2.17	0.0005	not found	not found
NMB1064	Q9JZG7	Putative uncharacterized protein	-	upregulated	2.41	0.0000	not found	not found
NMB1088	Q7DDI1	UPF0339 protein NMB1088	-	upregulated	2.19	0.0014	not found	not found
NMB1116	Q9JZC4	Putative uncharacterized protein	-	upregulated	2.37	0.0065	not found	not found
NMB1123	Q9JS68	Putative uncharacterized protein	-	upregulated	2.15	0.0039	not found	not found
NMB1151	Q9JS33	Sulfite reductase [NADPH] hemoprotein beta-component	cysI1	upregulated	6.12	0.0009	0004783; 0008652; 0016491; 0019344; 0046872; 0051536; 0055114	sulfur metabolism; amino acid biosynthesis; r
NMB1152	Q9JS45	Sulfite reductase [NADPH] flavoprotein alpha-component	cysJ1	upregulated	5.13	0.0005	0004783; 0006810; 0008652; 0010181; 0016491; 0019344; 0055114	sulfur metabolism; membrane transport; amin
NMB1153	Q9JRU7	Sulfate adenylyltransferase, subunit 1 (EC 2.7.7.4)	cysN-2	upregulated	5.13	0.0012	4781	sulfur metabolism
NMB1154	Q9JS34	Sulfate adenylyltransferase, subunit 2 (EC 2.7.7.4)	cysD-2	upregulated	4.95	0.0008	4781	sulfur metabolism
NMB1155	Q9JRT1	Phosphoadenosine phosphosulfate reductase (EC 1.8.4.8)	cysH1	upregulated	3.70	0.0032	0016491; 0019344; 0055114	redox; sulfur metabolism
NMB1156	P95370	Siroheme synthase	cysG1	upregulated	5.55	0.0010	0016491; 0055114	redox
NMB1207	P0A0R1	Bacterioferritin A (BFR A)	bfrA	upregulated	2.25	0.0245	0016491; 0046872; 0055114	redox; metal ion uptake
NMB1365	Q9JYZ0	Putative uncharacterized protein	-	upregulated	2.96	0.0002	not found	not found
NMB1369	Q9JYY6	Putative uncharacterized protein	-	upregulated	4.81	0.0001	not found	not found
NMB1370	Q9JYY5	Putative uncharacterized protein	-	upregulated	7.13	0.0006	not found	not found
NMB1371	Q9JYY4	Acetylornithine aminotransferase (ACOAT) (EC 2.6.1.11)	argD	upregulated	2.12	0.0007	8652	amino acid biosynthesis
NMB1378	P0A0Z0	Putative HTH-type transcriptional regulator NMB1378	-	upregulated	5.03	0.0000	not found	not found

continued (page 3/3)

Gene information				Transcriptome data			Functional annotation	
Gene ID	Uniprot ID	Gene product name	Symbol	Expression	FoldRatio (>2)	FDR (<0.1)	Enriched Gene Ontology (p<0.05)	functional group
NMB1379	Q9JYY0	Cysteine desulfurase (EC 2.8.1.7)	iscS	upregulated	4.84	0.0002	not found	not found
NMB1380	Q9JYX9	NifU protein	nifU	upregulated	5.68	0.0001	0016226; 0051536	iron-sulfur cluster
NMB1381	Q9JYX8	HesB/YadR/YfhF family protein	-	upregulated	5.58	0.0000	0005198; 0016226; 0051536	iron-sulfur cluster
NMB1386	Q4W567	Putative transposase	-	upregulated	2.09	0.0044	not found	not found
NMB1398	Q59623	Superoxide dismutase [Cu-Zn] (EC 1.15.1.1)	sodC	upregulated	2.09	0.0001	0004784; 0006801; 0016209; 0016491; 0046872; 0055114	redox; metal ion uptake
NMB1412	Q9JYV7	FrpC operon protein	-	upregulated	2.00	0.0014	not found	not found
NMB1414	Q9JYV6	FrpC operon protein	-	upregulated	2.02	0.0001	not found	not found
NMB1436	Q9JYT8	Putative uncharacterized protein	-	upregulated	2.30	0.0001	not found	not found
NMB1437	Q9JYT7	Uncharacterized protein NMB1437	-	upregulated	2.39	0.0005	30272	other functions
NMB1438	Q9JYT6	Putative uncharacterized protein	-	upregulated	3.32	0.0003	0016491; 0030272; 0051536; 0055114	redox; iron-sulfur cluster; other functions
NMB1463	not available	-	-	upregulated	2.05	0.0200	not found	not found
NMB1472	Q9JYQ8	Chaperone protein ClpB	clpB	upregulated	2.17	0.0072	9408	stress
NMB1475	Q9JYQ6	Putative uncharacterized protein	-	upregulated	12.70	0.0002	not found	not found
NMB1482	Q9JYQ0	Acyl CoA thioester hydrolase family protein	-	upregulated	2.12	0.0005	not found	not found
NMB1563	Q7DDA2	Transcriptional regulator, GntR family	-	upregulated	2.39	0.0000	5622	other functions
NMB1578	Q9JYH9	Putative uncharacterized protein	-	upregulated	2.36	0.0004	not found	not found
NMB1579	P64347	ATP phosphoribosyltransferase (ATP-PRT)	hisG	upregulated	2.67	0.0000	8652	amino acid biosynthesis
NMB1580	Q7DD99	Putative uncharacterized protein	-	upregulated	2.22	0.0075	not found	not found
NMB1590	Q9JYH0	Putative uncharacterized protein	-	upregulated	3.22	0.0050	0004601; 0016209; 0016491; 0055114	redox
NMB1719	Q9JY63	Efflux pump component MtrF	mtrF	upregulated	2.74	0.0005	not found	not found
NMB1753	Q9JY45	VapD-related protein	-	upregulated	3.83	0.0001	not found	not found
NMB1898	Q9JXT1	Lipoprotein	mlp	upregulated	2.65	0.0001	not found	not found
NMB1899	Q9JXT0	Putative uncharacterized protein	-	upregulated	2.52	0.0004	not found	not found
NMB1968	Q9JXM8	Aldehyde dehydrogenase A (EC 1.2.1.22)	aldA	upregulated	2.61	0.0009	0016491; 0055114	redox
NMB1984	not available	-	-	upregulated	2.26	0.0063	not found	not found
NMB1988	Q9JXL3	Iron-regulated outer membrane protein FrpB	frpB	upregulated	2.03	0.0037	0005215; 0006810	membrane transport
NMB2000	Q9JXK1	33 kDa chaperonin (Heat shock protein 33 homolog)	hslO	upregulated	2.25	0.0117	not found	not found
NMB2043	not available	-	#N/A	upregulated	2.18	0.0039	not found	not found
NMB2090	P0A0Y6	Phosphoheptose isomerase (EC 5.3.1.28)	gmhA	upregulated	2.01	0.0000	46872	metal ion uptake
NMB2141	Q7DD36	Putative uncharacterized protein	-	upregulated	2.25	0.0009	not found	not found
NMB0035	Q9K1P6	UPF0409 lipoprotein NMB0035	-	variable	2.08	0.0271	not found	not found
NMB0046	Q9K1N7	Putative uncharacterized protein	-	variable	2.06	0.0273	not found	not found
NMB0429	Q9K0X6	Putative uncharacterized protein	-	variable	2.38	0.0050	not found	not found
NMB0430	Q9K0X5	Putative carboxyphosphonoenolpyruvate phosphonomutase	-	variable	4.01	0.0030	not found	not found
NMB0431	Q7DDQ3	Methylcitrate synthase/citrate synthase 2 (EC 2.3.3.1)	prpC	variable	2.39	0.0009	0004108; 0006099; 0044262; 0046912	energy metabolism; other functions
NMB0546	Q9K0P0	Alcohol dehydrogenase, propanol-preferring (EC 1.1.1.1)	adhP	variable	4.54	0.0003	0016491; 0046872; 0055114	redox; metal ion uptake
NMB0634	P0A0Y4	Major ferric iron-binding protein (FBP) (Iron(III)	fbpA	variable	2.09	0.0127	0005215; 0006810; 0006811; 0046872	membrane transport; metal ion uptake
NMB0891	Q9JZU9	Putative uncharacterized protein	-	variable	2.48	0.0079	not found	not found
NMB1799	Q9JY09	S-adenosylmethionine synthase (AdoMet synthase)	metK	variable	2.28	0.0010	46872	metal ion uptake
NMB1844	Q9JXX8	Putative uncharacterized protein	-	variable	2.13	0.0366	not found	not found
NMB2096	Q9JXD7	Probable malate:quinone oxidoreductase (EC 1.1.5.4)	mqo	variable	2.13	0.0007	0006099; 0016491; 0055114	energy metabolism; redox

Supplementary Table S1B - Gene Ontology

Gene ontology information			Statistics		
**Gene Ontology**	**Name**	**functional group**	**# in dataset**	**# in genome**	**enrichment p-value**
0008652	cellular amino acid biosynthetic process	amino acid biosynthesis	10	62	0.01439
0009276	Gram-negative-bacterium-type cell wall	cell wall	3	9	0.02751
0006099	tricarboxylic acid cycle	energy metabolism	4	15	0.02348
0016226	iron-sulfur cluster assembly	iron-sulfur cluster	3	5	0.00580
0005198	structural molecule activity	iron-sulfur cluster	2	2	0.00930
0051536	iron-sulfur cluster binding	iron-sulfur cluster	6	32	0.02857
0006814	sodium ion transport	membrane transport	7	16	0.00017
0006810	transport	membrane transport	21	122	0.00018
0005215	transporter activity	membrane transport	10	50	0.00344
0006811	ion transport	membrane transport	7	27	0.00354
0005283	sodium:amino acid symporter activity	membrane transport	2	3	0.01997
0046872	metal ion binding	metal ion uptake	16	142	0.04899
0005622	intracellular	other functions	15	103	0.00741
0044262	cellular carbohydrate metabolic process	other functions	2	2	0.00930
0004108	citrate (Si)-synthase activity	other functions	2	2	0.00930
0030272	5-formyltetrahydrofolate cyclo-ligase activity	other functions	2	3	0.01997
0046912	transferase activity, transferring acyl groups	other functions	2	3	0.01997
0055114	oxidation-reduction process	redox	27	155	0.00002
0016491	oxidoreductase activity	redox	25	144	0.00004
0016655	oxidoreductase activity, acting on NADH or NADPH	redox	5	6	0.00008
0016209	antioxidant activity	redox	3	4	0.00313
0004784	superoxide dismutase activity	redox	2	2	0.00930
0006801	superoxide metabolic process	redox	2	2	0.00930
0010181	FMN binding	redox	3	8	0.02034
0004601	peroxidase activity	redox	2	4	0.03390
0022904	respiratory electron transport chain	redox	2	4	0.03390
0009408	response to heat	stress	2	3	0.01997
0019344	cysteine biosynthetic process	sulfur metabolism	4	4	0.00022
0008272	sulfate transport	sulfur metabolism	3	4	0.00313
0004781	sulfate adenylyltransferase (ATP) activity	sulfur metabolism	2	2	0.00930
0015419	sulfate transmembrane-transporting ATPase activity	sulfur metabolism	2	2	0.00930
0004783	sulfite reductase (NADPH) activity	sulfur metabolism	2	2	0.00930
0005840	ribosome	translation	14	57	0.00007
0030529	ribonucleoprotein complex	translation	13	56	0.00022
0003735	structural constituent of ribosome	translation	13	56	0.00022
0006412	translation	translation	15	100	0.00571
